# The effect and mechanisms of music therapy on the autonomic nervous system and brain networks of patients of minimal conscious states: a randomized controlled trial

**DOI:** 10.3389/fnins.2023.1182181

**Published:** 2023-05-12

**Authors:** Xiang Xiao, Wenyi Chen, Xiaoying Zhang

**Affiliations:** ^1^School of Music and Dance, Hunan First Normal University, Changsha, Hunan, China; ^2^School of Rehabilitation Medicine, Capital Medical University, Beijing, China; ^3^Department of Neurorehabilitation, China Rehabilitation Research Center, Beijing, China; ^4^Department of Music Artificial Intelligence and Music Information Technology, Central Conservatory of Music, Beijing, China; ^5^Music Therapy Center, China Rehabilitation Research Center, Beijing, China

**Keywords:** disorders of consciousness, minimally consciousness state, music therapy, music auditory stimulation, autonomic nervous system, fMRI-DTI

## Abstract

**Introduction:**

Music therapy has been employed as an alternative treatment modality for the arousal therapy of patients with disorders of consciousness (DOC) in clinical settings. However, due to the absence of continuous quantitative measurements and the lack of a non-musical sound control group in most studies, the identification of the specific impact of music on DOC patients remains challenging. In this study, 20 patients diagnosed with minimally consciousness state (MCS) were selected, and a total of 15 patients completed the experiment.

**Methods:**

All patients were randomly assigned to three groups: an intervention group (music therapy group, *n* = 5), a control group (familial auditory stimulation group, *n* = 5), and a standard care group (no sound stimulation group, *n* = 5). All three groups received 30 min of therapy five times a week for a total of 4 weeks (20 times per group, 60 times in total). Autonomic nervous system (ANS) measurements, Glasgow Coma Scale (GCS), and functional magnetic resonance—diffusion tensor imaging (fMRI-DTI) were used to measure the peripheral nervous system indicators and brain networks, and to evaluate patients’ behavior levels.

**Results:**

The results reveal that PNN50 (*p* = 0.0004**), TP (*p* = 0.0003**), VLF (*p* = 0.0428**), and LF/HF (*p* = 0.0001**) in the music group were significantly improved compared with the other two groups. Such findings suggest that the ANS of patients with MCS exhibits higher activity levels during music exposure compared to those exposed to family conversation or no auditory stimulation. In fMRI-DTI detection, due to the relative activity of ANS in the music group, the ascending reticular activation system (ARAS) in the brain network also exhibited significant nerve fiber bundle reconstruction, superior temporal gyrus (STG), transverse temporal gyrus (TTG), inferior temporal gyrus (ITG), limbic system, corpus callosum, subcorticospinal trace, thalamus and brainstem regions. In the music group, the reconstructed network topology was directed rostrally to the diencephalon’s dorsal nucleus, with the brainstem’s medial region serving as the hub. This network was found to be linked with the caudal corticospinal tract and the ascending lateral branch of the sensory nerve within the medulla.

**Conclusion:**

Music therapy, as an emerging treatment for DOC, appears to be integral to the awakening of the peripheral nervous system-central nervous system based on the hypothalamic-brainstem-autonomic nervous system (HBA) axis, and is worthy of clinical promotion. The research was supported by the Beijing Science and Technology Project Foundation of China, No. Z181100001718066, and the National Key R&D Program of China No. 2022YFC3600300, No. 2022YFC3600305.

## Introduction

Minimal consciousness state (MCS) is a serious disorder of consciousness (DOC), which is different from vegetative state (*VS*) (Eapen et al., 2017). MCS is primarily characterized by a patient’s capacity to exhibit limited yet distinct self-awareness and environmental perception (Rasmus et al., 2019). In 2022, the International Society of Disorders of Consciousness defined MCS as “a state with small and clear behavioral evidence of severe conscious changes in the perception of the self and the environment”(Bodien et al., 2022; Bower et al., 2022). Studies have shown that patients with MCS have a relatively complete neural network under severe brain damage, which differs from persistent *VS* (Fischer et al., 2022; Fitzpatrick-DeSalme et al., 2022; Kondziella and Stevens, 2022). Neurobehavioral and imaging studies have revealed that there are significant differences in clinical manifestations and neurological symptoms between MCS and *VS* (Istace, 2022). However, owing to the variability of arousal levels and the dysfunction of sensory, motor, and language systems in patients with DOC, communication between patients and examiners is restricted, leading to a high incidence of misdiagnosis in clinical practice (Bellon et al., 2022; Ismail et al., 2022). At the same time, MCS has greater neurological rehabilitation potential than *VS* patients in terms of prognosis, and thus, is of great clinical value for multiple wake-promoting treatments for MCS patients (Bagnato, 2022).

The DOC patient assessment determines the level of awareness of a patient by identifying whether the response to the stimulus is reflexive, or comes from an active action in which part of the perceptual capacity is engaged (Young and Peterson, 2022). The current clinical assessment scales for early disturbance of consciousness include Glasgow Coma Scale (GCS) (Mehta and Chinthapalli, 2019), Coma Recovery Scale-revised (CRS-R) and other scales, which are used to differentiate *VS* from MCS (Giacino et al., 2004). Moreover, neuroimaging assessment is also a significant tool for the diagnosis of DOC. Structural imaging techniques, including T1 and T2 weighted magnetic resonance imaging (MRI) and functional MRI (fMRI) (Sanz et al., 2021), can facilitate the quantification of brain atrophy in patients with DOC (Fins, 2011). Such methods can also effectively identify the precise location of brain injuries, hypoxic–ischemic lesions, and diffuse axonal injuries (Humble et al., 2018). Fractional anisotropy (FA) of key regions detected by diffusion tensor imaging (DTI) is a reference index for predicting the prognosis of DOC (Li et al., 2022). In general, CRS-R is used as the preferred tool for prognosis evaluation in clinical practice, and GOS-E is used as an auxiliary scale for prognosis evaluation (Annen et al., 2019).

At present, DOC lacks exact and effective treatment methods. Despite the lack of systematic research and sufficient evidence-based medical evidence, clinical research and attempts to treat DOC have been conducted in consideration of the large number of DOC patients and the considerable treatment demand. The main treatment methods include surgery, medication, hyperbaric oxygen therapy, neuromodulation therapy (invasive, non-invasive, etc.), physical therapy, and others. As an emerging rehabilitation modality in recent years, music therapy has shown significantly positive effects in promoting recovery for patients with consciousness disorders (Grimm and Kreutz, 2018; Bower et al., 2022; Liu et al., 2022). Music has a wide range of activation effects on the cerebral cortex, such as the bilateral frontal lobe, temporal lobe, parietal lobe and cerebellum, and the emotion-related frontal lobe, cingulate gyrus, amygdala and hippocampus are particularly responsive (Rollnik and Altenmüller, 2014). The use of a patient’s favorite music for auditory stimulation is conducive to the recovery of consciousness (Magee et al., 2015).

Music, particularly songs that patients find appealing, frequently elicit emotional resonance (Hu et al., 2021). One vital strategy in accomplishing music therapy is to select songs that patients enjoy and have them performed live by music therapists (Magee et al., 2014). The songs sung by the music therapist in the present study were chosen accurately based on the patients’ preferences and emotional engagement (Pool et al., 2020). The effects of music intervention, including both active and receptive music therapy, had a positive impact on patients with DOC (Zhang et al., 2021). In the field of rehabilitation medicine, awakening treatment of DOC combined with music therapy, as a different treatment strategy, can effectively improve the consciousness state of patients with cerebral MCS (Arroyo-Anlló et al., 2013). Evidence has shown that music can activate the function of the default mode affective network in patients with DOC (Janelli et al., 2004; Riganello et al., 2015; De Luca et al., 2022). Despite such findings, there are difficulties in terms of drawing conclusions due to the few studies that did not use continuous quantitative measures and the lack of control groups to show the distinctive effects of music on patients with DOC. Three groups were used to compare the activation of autonomic nervous system and brain network in patients with MCS over 4 weeks: music therapy group (played by music therapist), the voices of family members (recorded), and a standard care group, to clarify the specific role of music therapy.

## Subjects and methods

Twenty patients were recruited from China Rehabilitation Research Center, and the inclusion criteria were as follows: (1) MCS diagnosed by CRS-R and GOS-E (Giacino et al., 2004); (2) inpatients with a disease duration of at least 3 months; (3) aged 18–60 years old; (4) can tolerate the treatment in the supine position for more than half an hour; (5) no previous music education background; (6) informed consent was obtained from patients and their families. The exclusion criteria consisted of: (1) severe arrhythmia, malignant arrhythmia or other diseases, or history of cardiac surgery; (2) orthostatic hypotension; (3) severe hearing impairment. The withdrawal and termination criteria were as follows: termination of treatment can occur if the patient’s condition changes due to discharge or voluntary withdrawal. A total of 15 subjects completed the experiment. Five subjects (*n* = 5) withdrew from the experiment due to transfer to another hospital or personal reasons. The characteristic data are presented in Table 1.

**Table 1:** Baseline characteristics of participants included in the present study.

	Intervention group	Control group	Standard care	Value of *p*
	Mean ± SD	Mean ± SD	Mean ± SD	
Minimally consciousness state	5	5	5	>0.05
(MCS)				
Gender				
Male	2	3	4	>0.05
Female	3	2	1	>0.05
Age	26.8 ± 11.21	50.8 ± 10.13	38.8 ± 15.07	
Time since injury	7.53 ± 5.04	5.79 ± 4.41	8.15 ± 1.33	
Education background				
primary school	1	3	0	>0.05
junior high school	0	1	2	>0.05
Bachelor degree or above	4	1	3	>0.05

Patient recruitment was conducted from December 2019 to September 2022, the data of participants’ characteristics are shown in Table 1. Fifteen patients were randomly divided into three groups: the intervention group was the music therapy group (*n* = 5), the control group was the familial auditory stimulation group (*n* = 5), and the standard care group was the no auditory stimulation intervention group (*n* = 5). There were no significant differences in the ratio of male to female, age, time of injury and education among the three groups (*p* > 0.05).

Table 1 presents the distribution of group differences, gender, age, injury time and education background of the three groups of patients. The remaining data were expressed as standard deviation + mean and analyzed by paired t test. Experimental group: music therapy group; Control group: familial auditory stimulation group; standard care: standard care group. *p* > 0.05 indicates that there was no significant difference among the three groups.

### Study design

The present study constituted a randomized controlled trial with a pre-and post-test experimental design that featured three separate groups, namely the experimental (*n* = 5), control (*n* = 5), and standard care group (*n* = 5). The study was conducted using a single-blind design, in which participants only knew they were participating in a clinical trial after signing an informed consent form, and a masking design was used for grouping information and data analysis. The study was conducted at CRRC from December 2019 to November 2022. This study was supported by the Beijing Science and Technology Project Foundation of China, No. Z181100001718066; and the National Key R&D Program of China No. 2022YFC3600300, No. 2022YFC3600305. This research proposal has been approved by the Ethics Committee of CRRC (approval No. 2018–022-1) on March 12, 2018 (Supplementary material), and informed consent (Supplementary material) was obtained from the participants, relatives or guardians before commencing the study. The study trial was registered with the Clinical Trial Registry (Registration No. ChiCTR1800017809) on August 15, 2018.

### Procedure

**Figure 1:**
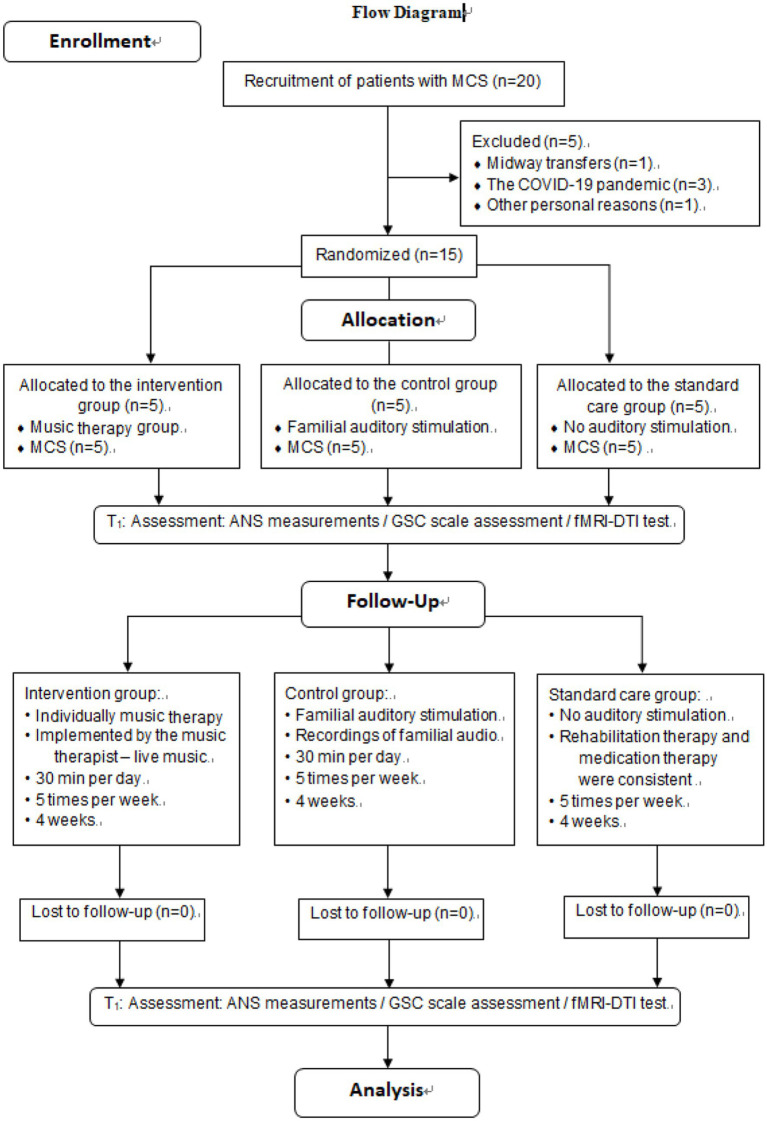
Flow diagram, consort flowchart for participants’ recruitment and allocation.

After approval by the CRRC Ethics Committee and registration for clinical trials, subjects were initially screened by neurosurgeons. Patients who initially received a score on the GCS indicating moderate to severe impairment of consciousness were referred to the music therapy department following consultation. Potential participants were identified by music therapy investigators according to predetermined inclusion and exclusion criteria. Subsequently, the investigators confirmed the eligibility of the patients and invited their family members to participate in the study after obtaining their signed informed consent forms. Topics included study purpose, procedure, risks, benefits, confidentiality, and subjects’ rights. Upon enlistment, GCS scores were used to determine whether patients had impaired hearing function and computer-generated sequences (Excel 2013, Microsoft Office, Seattle, WA, USA) were used to randomly assign patients to one of the three groups. Participants in the intervention group received music therapy from a music therapist for 4 weeks, while participants in the control group received familial auditory stimulation for 4 weeks, with no acoustic stimulation in the standard care group. The enrollment and assignment of participants is shown in Figure 1.

Figure 1 illustrates that 20 participants were enrolled in the study, 15 participates completed the trial, and 5 participants withdrew: COVID-19 (*n* = 3), midway transfers (*n* = 1) or other personal reasons (*n* = 1). The experimental group (*n* = 5) received music therapy administered by a music therapist, whereas the control group (*n* = 5) was subjected to auditory stimulation provided by their respective family members. Meanwhile, the standard care group was given no auditory stimulation. Two rounds of evaluation were conducted during the whole period, namely T0 (baseline) and T1 (after 4 weeks). The data analysis included a sample of 15 patients who were in a minimally conscious state.

### Interventions

The treatment intervention began after the subjects were enrolled. All patients in the experimental group received live music therapy the control group received sound auditory stimulation recorded by family members, and standard care group did not receive auditory stimulation. Each patient received 30 min of training five times a week for 4 weeks. Music therapy was performed by registered music therapists who were licensed to ensure the professionalism of the intervention. The familial stimuli were generated through recordings of family member voices, with the family members engaging in conversation pertaining to the patient’s past personal life experiences, and expressing such content directly to the patient.

#### The live music therapy supported by music therapist

In the intervention group, a music therapist performed a fixed program of musical therapy session (a set of songs edited in a fixed order). The song selection was based on the patient’s musical preferences. Sources of songs are: (1) Songs that are played most frequently in the mobile phone music app of patients; (2) The family members informed the music therapist of the patient’s favorite singer, and the music therapist selected the most famous songs of the singer for intervention. The standardized procedure for music therapy consisted of a fixed pattern lasting for a duration of 30 min, comprising (1) an opening piece, specifically the “Hello song” composed by the therapist with a duration of 2.5 min, followed by (2) a song content component that involved the selection of music with emotional significance to the subjects and their significant relationships (for example, parents, couples/lovers, children, grandparents, friends) from their past life experiences, with a total duration of 25 min. For instance, one subject had previously viewed the film “Hello, Li Huanying” with their mother before experiencing injury, and had a powerful emotional response to the theme song “Daylily Flower” played at the end of the movie. As a result, during the music sessions, the music therapist performed the song live for the patient, while simultaneously incorporating a section of lyrics that were thematically related to the mother based on the film’s premise. The music therapy procedure concluded (3) with the “Goodbye Song” (2.5 min), which featured lyrics composed by the therapist (Supplementary material).

#### Familial auditory stimulation supported by patients’ family members

The researchers communicated with the patients’ families to confirm the content and duration of the recordings. The recording content was related to the patients’ personal life experiences. The patients’ loved ones had a daily conversation around the theme of each “patient’s personal life,” and the conversation content was recorded in WMA or MP3 format for 30 min (Supplementary material).

#### Standard care group

The standard care group did not receive auditory stimulation, but the rehabilitation therapy and medication therapy were consistent with the experimental group and the control group.

### Measurements

Before the intervention, all the participants were assessed at baseline by the researcher, using (1) automatic nerve system (ANS) testing for physiological examination of the peripheral nervous system; (2) the Glasgow Coma Scale (GCS) (Mehta and Chinthapalli, 2019) for behavioral assessment; and (3) Functional Magnetic Resonance Imaging-Diffusion Tensor Imaging (fMRI-DTI) (Gould et al., 2021). Behavioral and radiological changes were observed at a second assessment 4 weeks later.

### Automatic nerve system test

The ANS Bodyguard device (Version 3.1, Meiyang Limited, Beijing, China) was used to record the sympathetic and parasympathetic nervous system indicators as follows: (1) Percentage of the number of adjacent sinus beats with difference > 50 ms in total sinus beats, PNN50; (2) Total Power, TP; (3) Low Frequency, LF; (4) High Frequency, HF; (5) LF norm/HF norm = LF/(LF + HF) × 100; (6) Very low frequency band, VLF. Low frequency and high frequency were obtained in the ranges of 0.04–0.15 Hz and 0.15–0.40 Hz, respectively (Gitler et al., 2022). The magnitude of high frequency and the ratio of low frequency to high frequency (LF/HF) corresponded to the intensity of vagal activity and sympathetic vagal balance, respectively. Specifically, the size of LF is involved in vagus and sympathetic nerve activity (Lee and Shields, 2022). The natural logarithms of powers (lnLF and lnHF) were used to evaluate the magnitude of each spectral component. The ratio of LF component to HF component (LF/HF ratio) was calculated by dividing lnLF by lnHF (lnLF/lnHF).

### Glasgow coma scale (GCS)

GCS is a behavioral assessment method for assessing the degree of consciousness of patients (Eapen et al., 2017). The level of consciousness was assessed by evaluating eye opening, language, and movement. A higher score indicated a better state of consciousness. Specifically (1) the blink reflex was graded on a scale of self-opening (4′), opening eyes upon hearing one’s name (3′), opening eyes in response to a painful stimulus (2′), or no reaction (1′); (2) the language reflex was graded on a scale of accurate orientation and correct responses (5′), correct orientation but incorrect responses (4′), ability to speak but unable to answer (3′), ability to produce only sounds (2′), and inability to produce any sounds (1′); and (3) the motor reflex was graded on a scale of ability to follow commands (6′), ability to point to the site of pain (5′), retraction of limbs in response to a painful stimulus (4′), flexion of both upper limbs (3′), extension of limbs (2′), and complete relaxation of limbs (1′) (Mehta and Chinthapalli, 2019).

### Functional magnetic resonance imaging-diffusion tensor imaging

Functional Magnetic Resonance Imaging (fMRI) uses magnetic resonance imaging to measure the hemodynamic changes caused by neuronal activity. Generally, fMRI has the scanning characteristics of high spatial resolution (2-3 mm) and high temporal resolution (within 1 s, rapid imaging time is 30-100 mm). Such method can reveal the functional reorganization of different regions of the brain. Diffusion tensor imaging (DTI), a type of fMRI technique that examines the connectivity and integrity of living tissue, is designed to visualize the direction of nerve fiber bundles in the white matter of the brain, resulting in a detailed tensor image of the central nervous system fibers.

The DTI index adopted in the present study was a parameter of fractional anisotropy (FA) of the reaction part. The value ranged from 0 to 1, where 0 represents the maximum anisotropic dispersion and 1 represents the maximum anisotropic dispersion. The method involved sensitive gradients applied in six different non-collinear directions with a slice thickness of 1 mm, using a 256 matrix scanning protocol and a 256 × 256 isotropic resolution of 1 square mm. Scans were performed at T1 and T2 to visualize the fiber tracts of water molecules in the X, Y, and Z directions of the brain.

### Statistical analysis

Measurements of the three groups were collected at 2 time points before intervention (baseline, T0) and after intervention (4 weeks later, T2), being expressed in the form of mean ± standard deviation. Two-factor analysis of variance was used to observe the differences between differences groups, time effects, and the interaction effects between time and groups. SPSS 23.0 (SPSS Inc., Chicago, IL, USA, IBM Lenovo, BJIBM Lenovo, BJ, USA) was used for statistical analysis of the three sets of data to determine the specific effects of the intervention.

## Results

### The ANS index in the intervention group was significantly improved compared with the control group and the standard care group

**Figure 2:**
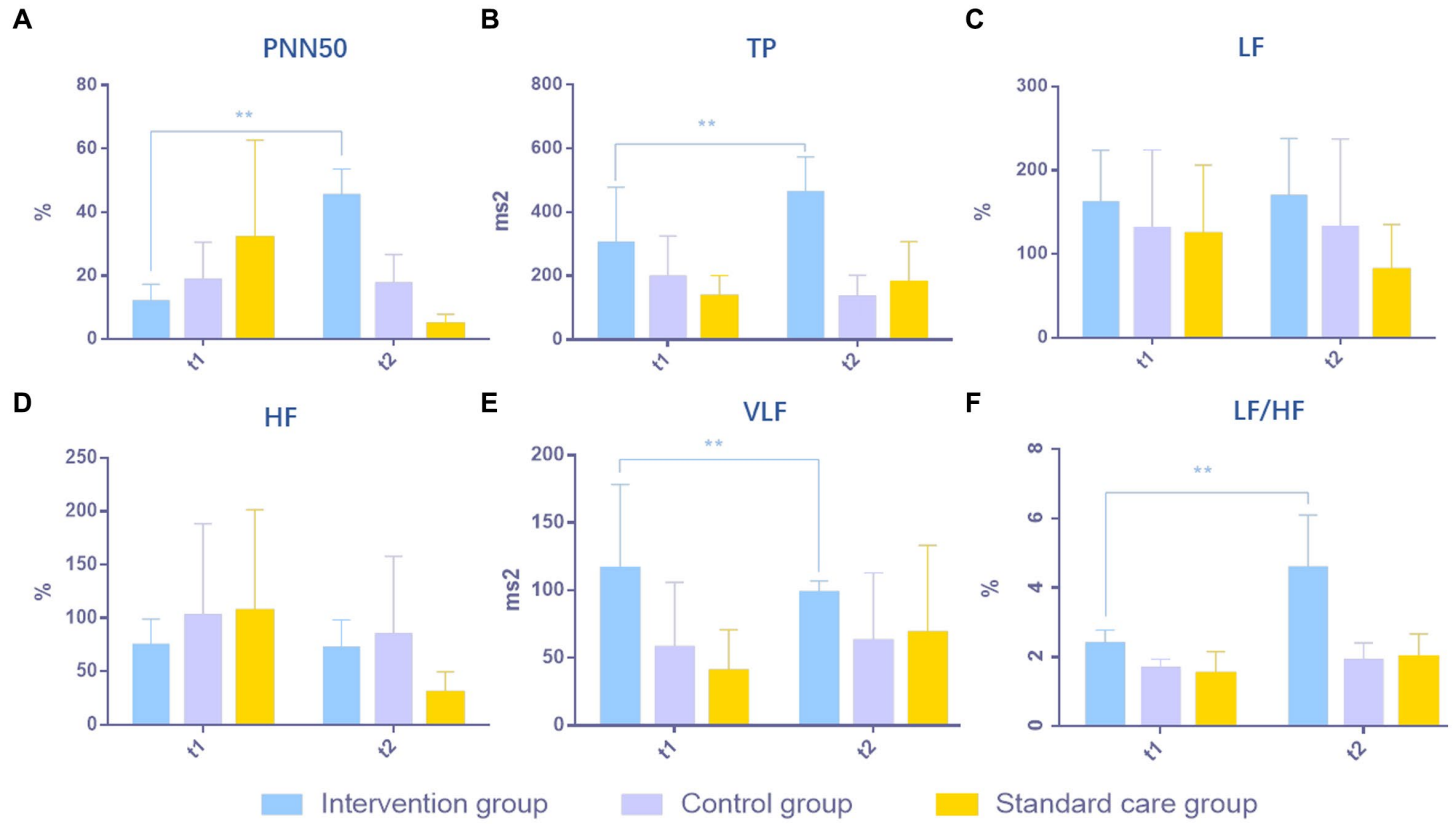
Comparison of results of ANS in MCS patients in three groups. **(A)** PNN50, **(B)** TP, **(C)** LF, **(D)** HF, **(E)** VLF, **(F)** LF/HF. **p < 0.01, significant difference; *p < 0.05, difference.

The excitability of the ANS system in MCS patients was evaluated in respect of six aspects: (1) PNN50; (2) TP; (3) LF; (4) HF; (5) VLF; and (6) LF/HF. After 4 weeks of treatment, PNN50 was significantly higher in the intervention group (a) than in the control group (b) and the standard care group (c) (*p* = 0.0004, a > b > c, Figure 2A). TP in the intervention group was significantly higher than those in the control group and the standard care group (*p* = 0.0003, a > b > c, Figure 2B). For LF, there were no obvious differences between the three groups (*p* = 0.2401, a≒b≒c, Figure 2C), which was the same for HF (*p* = 0.1685, a≒b≒c, Figure 2D). VLF in the intervention group was significantly higher than those in the control group and the standard care group (*p* = 0.0428, a > b > c, Figure 2E). However, for the ratio of LF/HF, the intervention group showed considerably significant improvement over the control group and the standard care group (*p* = 0.0001, a > b > c, Figure 2F). The results are shown in Table 2 and Figure 2.

**Table 2:** The results of the ANS tests in patients with minimally conscious states across the study period for the intervention group (a), the control group (b), and the standard care group (c).

		Intervention group (a)	Control group (b)	Standard care (c)	*p*	Intergroup comparison
		(*n* = 5)	(*n* = 5)	(*n* = 5)		
		Mean ± SD	Mean ± SD	Mean ± SD		
PNN50 (%)	*t*_1_	11.92 ± 5.40	18.68 ± 11.91	31.89 ± 30.78	0.7475	a>b>c **
	*t*_2_	45.39 ± 8.25	17.58 ± 9.10	4.73 ± 3.15	0.0004**	
TP (ms^2^)	*t*_1_	303.01 ± 175.37	196.74 ± 128.15	135.62 ± 65.06	0.2903	a>b>c **
	*t*_2_	462.61 ± 110.49	134.07 ± 68.07	179.38 ± 128.13	0.0003**	
LF (%)	*t*_1_	161.67 ± 62.15	130.79 ± 93.49	123.92 ± 82.53	0.7436	a≒b≒c
	*t*_2_	169.20 ± 68.99	132.45 ± 105.01	80.95 ± 54.33	0.2401	
HF (%)	*t*_1_	74.73 ± 24.38	102.59 ± 85.88	106.75 ± 94.90	0.3901	a≒b≒c
	*t*_2_	72.18 ± 26.17	84.63 ± 73.11	30.15 ± 19.48	0.1685	
VLF (%)	*t*_1_	116.14 ± 62.27	57.36 ± 48.62	40.04 ± 30.91	0.5712	a>b>c *
	*t*_2_	98.04 ± 8.99	62.44 ± 50.52	68.28 ± 64.92	0.0428*	
LF/HF (%)	*t*_1_	2.39 ± 0.39	1.68 ± 0.26	1.52 ± 0.64	0.0191	a>b>c **
	*t*_2_	4.58 ± 1.52	1.91 ± 0.50	1.99 ± 0.68	0.0001**	

Data were expressed as mean ± SD (*n* = 15), and two-way ANOVA was used to analysis the data. **p < 0.01, significant difference; *p < 0.05, difference. >, greater than or equal to; ≒, approximately equal to.

### The GCS scores in the intervention group were significantly improved compared with the control group and the standard care group

**Figure 3:**
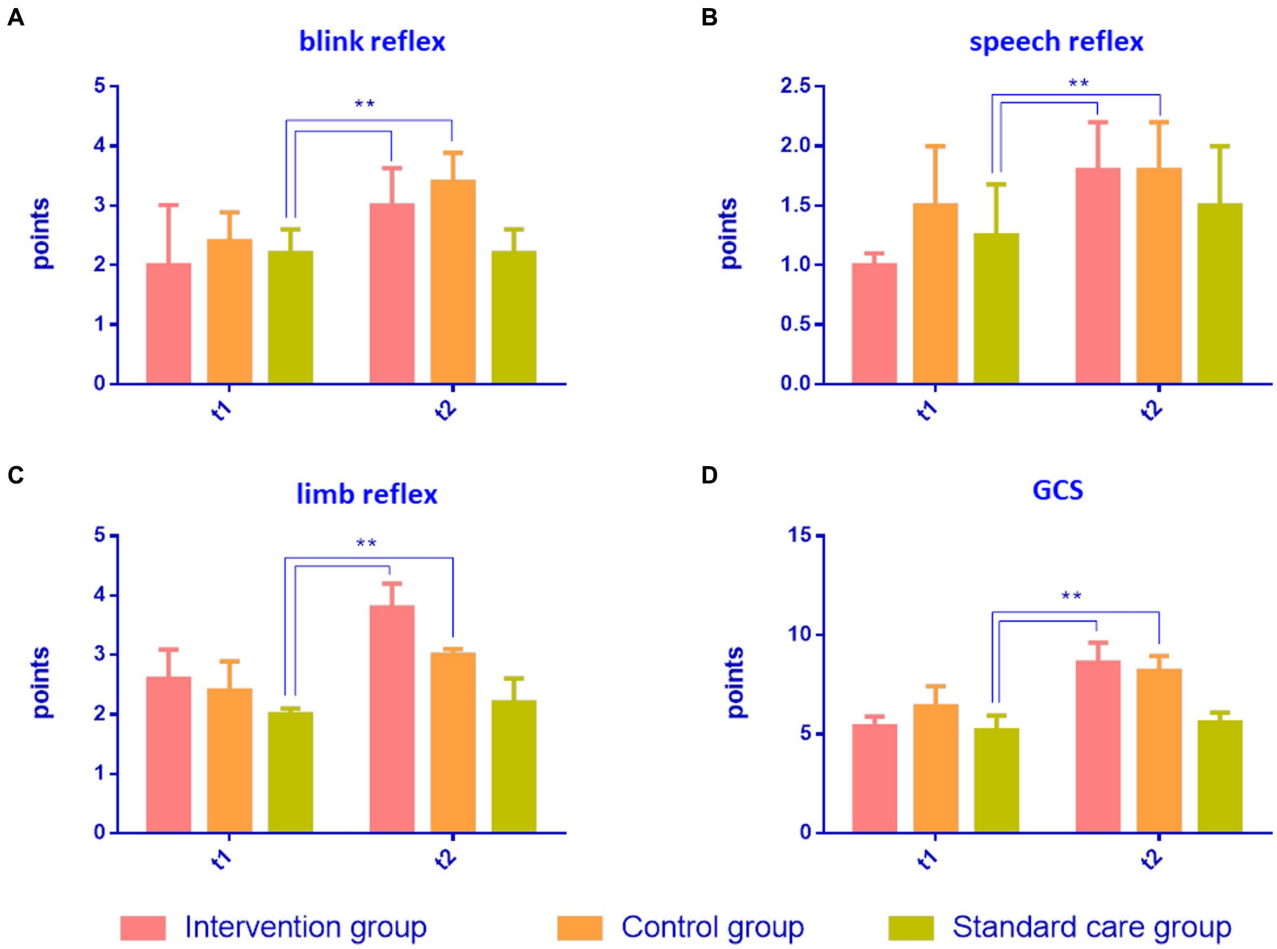
Comparison of results of GCS scores in MCS patients in three groups. **(A)** Blink reflex, **(B)** speech reflex, **(C)** limb reflex, and **(D)** GCS score. **p < 0.01, significant difference; *p < 0.05, difference.

The degree of consciousness of MCS patients was scored using the GCS. GCS can be evaluated in four domains: (1) blink reflex; (2) speech reflex; (3) limb reflexes, and (4) total GCS score. After 4 weeks of treatment, the blink reflex frequencies in the intervention group (a) and control group (b) were significantly higher than that in standard care group (c), and the score in the intervention group was higher than that in the control group (*p* = 0.0071, a > b > c, Figure 3A). The speech reflex frequencies of the intervention group and the control group were significantly higher than that of the standard care group (c), and the score of the intervention group was higher than that of the control group (*p* = 0.0063, a > b > c, Figure 3B). The limb reflex frequencies were significantly higher in the intervention and control groups than in the standard care group (c), where the intervention group scored higher than the control group (*p* = 0.0001 a > b > c, Figure 3C). The total GCS scores of the intervention and control groups were higher than that of the standard care group (c), where the intervention group had a higher score than the control group (*p* = 0.0001, a > b > c, Figure 3D). The results are shown in Table 3 and Figure 3.

**Table 3:** The results of GCS scores in patients with minimally consciousness states across the study period for the intervention group (a), the control group (b), and the standard care group (c).

		Intervention group (a)	Control group (b)	Standard care (c)	*p*	Intergroup comparison
		(*n* = 5)	(*n* = 5)	(*n* = 5)		
		Mean ± SD	Mean ± SD	Mean ± SD		
Blink	*t*_1_	2.02 ± 0.01	2.40 ± 0.49	2.20 ± 0.40	0.3084	
	*t*_2_	3.01 ± 0.63	3.40 ± 0.49	2.20 ± 0.40	0.0071**	a > b > c**
Speech	*t*_1_	1.01 ± 0.01	1.05 ± 0.50	1.25 ± 0.43	0.274	
	*t*_2_	1.80 ± 0.40	1.80 ± 0.40	1.50 ± 0.50	0.0063**	a > b > c**
Motor	*t*_1_	2.60 ± 0.49	2.40 ± 0.49	2.01 ± 0.01	0.784	
	*t*_2_	3.80 ± 0.40	1.50 ± 0.50	2.20 ± 0.40	0.0001**	a > b > c**
Total	*t*_1_	5.40 ± 0.49	6.40 ± 1.02	5.20 ± 0.75	0.2379	
	*t*_2_	8.60 ± 1.02	8.20 ± 0.75	5.60 ± 0.49	0.0001**	a > b > c**

Data were expressed as mean ± SD (*n* = 15), and two-way ANOVA was used to analysis the data. **p < 0.01, significant difference; *p < 0.05, difference. >, greater than or equal to.

### Visualization effect of music therapy on brain network in the patients with MCS

**Figure 4:**
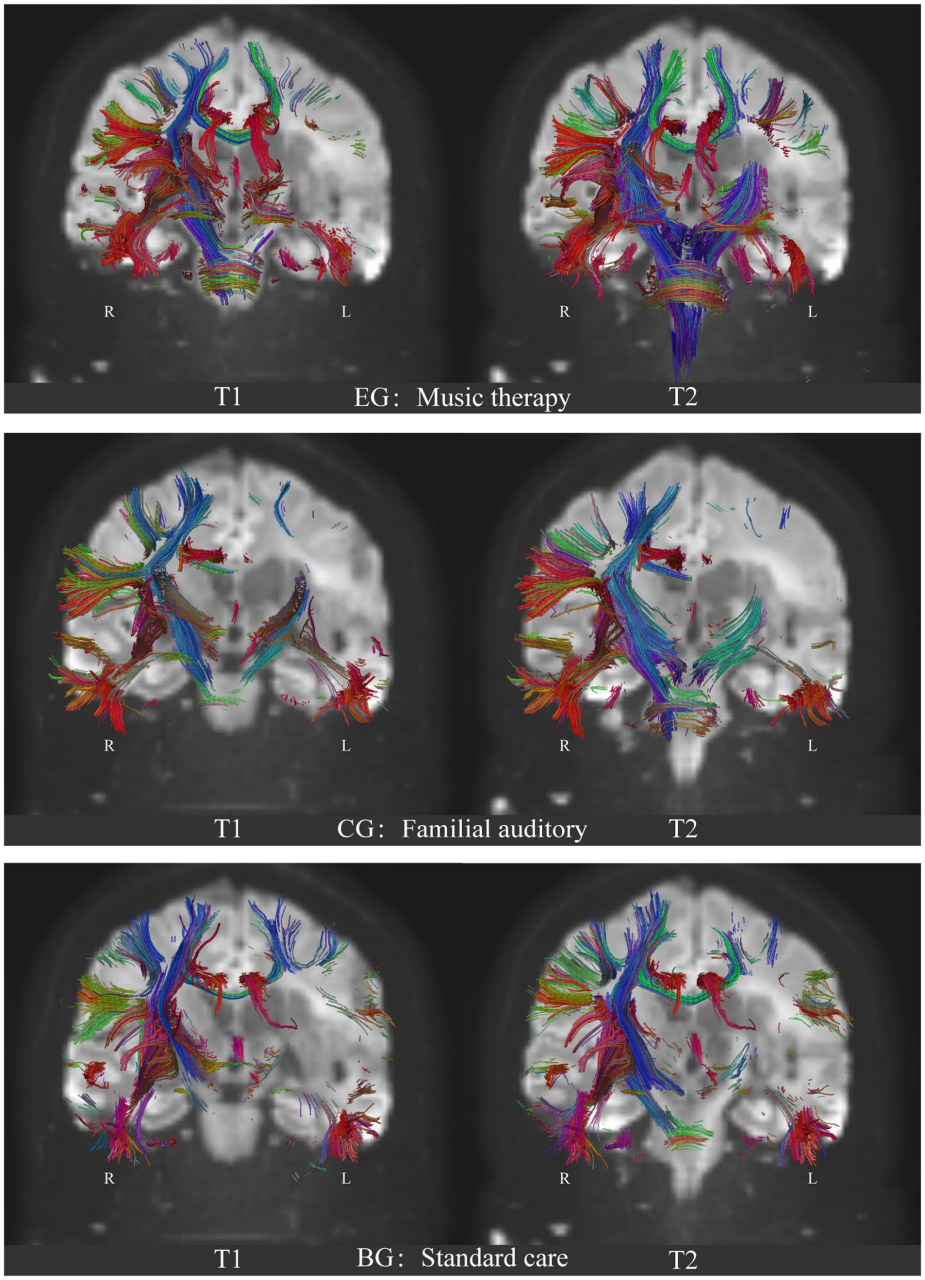
Comparison of the neural fibers trace results of DTI on brain network of the three groups. EG: experimental group, music auditory stimulation group; CG: control group, familial auditory stimulation group, BG: standard care group, no auditory stimulation group. T1: baseline, before intervention; T2: 1 month after intervention. R: right hemisphere; L: Left hemisphere. Blue: Superior–Inferior direction of the nerve fiber bundle; Red: Anterior–Posterior direction of the nerve fiber bundle; Green: Left–Right direction of nerve fiber bundles.

After performing Pipeline for Analyzing braiN Diffusion imAges (PANDA) analysis using MATLAB and considering the 246 brain regions (Fan et al., 2016), it was observed that compared with the control group, the dorsolateral area of the superior frontal gyrus (SFG), middle frontal gyrus (MFG), ventrolateral of middle frontal gyrus, orbitofrontal cortex, precentral gyrus, superior temporal gyrus, transverse temporal gyrus, inferior temporal gyrus, corpus callosum, parahippocampal, inferior parietal lobule, postcentral gyrus, insular gyrus, cingulate gyrus, basal ganglia and other regions exhibited a significant increase in Fractional Anisotropy (FA), Fiber Number (FN), and Path Length (Length). Table 3 shows the highlighted regions of interest (ROIs) of FA, FN, and length enhancement trends after music-based MIT intervention (Figure 4; Supplementary material).

### Positive effects of music therapy on hypothalamic-brainstem-autonomic nervous system axis of autonomic nervous system-central nervous system in patients with MCS

After PANDA analysis using MATLAB and considering the accuracy of white matter fiber bundle shape tracking by DTI (Fan et al., 2016), it was identified that compared with the control group and the standard care group, neural fiber traces in the superior frontal gyrus (SFG), middle frontal gyrus (MFG), precentral gyrus (PrG), postcentral gyrus (PoG), superior temporal gyrus (STG), transverse temporal gyrus (TTG), inferior temporal gyrus (ITG), limbic system, corpus callosum, subcorticospinal trace, thalamus and brainstem regions were significantly increased in the experimental group. Figure 4 shows the results of the experimental group after music therapy compared with the control group and the standard care group. The results of the three groups are shown in Figure 4.

## Discussion

By autonomously regulating the nervous system according to the different auditory stimuli given, music can widely activate the brain network (Zatorre et al., 2007; Alluri et al., 2012; Särkämö et al., 2013; Koelsch, 2014; Fan et al., 2016) and increase the blood flow of intracerebral arteries, thereby providing a favorable environment for the overall recovery of the brain. The auditory complexity of music has an environmental enrichment effect (Engineer et al., 2004; Teppo et al., 2008) in patients with MCS, which has a behavioral and neurobiological level of facilitation in autonomic nervous system arousal in patients with DOC. Several prior studies have reported on neural reorganization of brain networks in patients with MCS following music-supported therapy. Additionally, other studies have provided further evidence of music therapy on central neural plasticity related to auditory and motion (Amengual et al., 2013; Grau-Sanchez et al., 2013; Ripollés et al., 2016). The aim of the present study was to focus on two key points: firstly, investigating the activity of the autonomic nervous system based on the hypothalamus-pituitary–adrenal axis in MCS patients stimulated by music. Secondly, observing the structural remodeling outcomes of neural networks in the brain when the peripheral nervous system is activated.

### The ANS activity of MCS patients was increased in music therapy—based on the theory of hypothalamic brainstem autonomic nervous system

Music produces measurable cardiovascular and endocrine responses, indicated by reduced serum cortisol levels and inhibition of cardiovascular stress reactions (Sihvonen et al., 2017). This theory relies on the musical effectiveness on the hypothalamic brainstem autonomic (HBA) axis, which is proposed in 2017, which states that music-induced activation of the parasympathetic nervous system and inhibition of the sympathetic nervous system, and induce improvement of arousal might therefore enhance recovery of cognitive functions in patients (Sihvonen et al., 2017). In the regulation of the autonomic nervous system (ANS), external stimuli are received through bodily receptors, serving as a crucial source for the modulation of the sympathetic and parasympathetic nervous systems. The autonomic nervous system is often referred to as the involuntary nervous system due to its lack of control by conscious awareness. In the control of ANS, signals from the outside world are received through body receptors, which is a significant stimulus source for the antagonism or inhibition of ANS sympathetic and parasympathetic nerves. In the present study, patients in the experimental group received music therapy through auditory means. The song selection was based on the patients’ personal life experiences and included their favorite songs prior to the onset of illness. The input form was auditory stimulation in the form of live singing of the songs by a music therapist. Patients in the music group received more varied sound signals than those in the control group who listened to family voices and the standard care group who received no auditory stimuli. Compared with the abstract content of familial conversation (better brain function is needed to recognize sound properties and understand the meaning of the conversation), the emotional experience brought by the melodic and harmonic richness of music is more concentrated in the thalamus and hypothalamus, which are the central starting point of the HBA axis. As such, after receiving auditory input from music, PNN50 and TP in the ANS in the music group were significantly increased. Decreased PNN50 indicates decreased parasympathetic excitability (Tak et al., 2010). Since patients in the standard care group were not treated with auditory stimulation other than standard care and nutritional neuro-medications, parasympathetic PNN50 decreased compared with patients in the other two groups who received auditory interventions, suggesting the possibility of autonomic nervous system disorders. Such findings indicate that while listening to the familiar music that made patients feel present, the experimental group exhibited an increase in sinus heart rate, myocardial sympathetic and vagal tone, as well as a decrease in balance. When the experimental group listened to familiar music, significant changes in VLF, LF/HF and other values of ANS showed that the experience generated during listening to music could induce immediate cardiovascular and endocrine responses, and such positive experiences were related to the fast reward circuit. According to the upward circulation of the HBA axis, after stimulation by music, ANS excitation could further result in activity of the brain stem and hypothalamus, thereby promoting the activity of the MCS brain network.

### Music therapy activates the brain network or spare neural networks more broadly in MCS patients—based on the results of DTI analysis

According to the results of FA (anisotropy) in the analysis of fMRI-DTI, the FA showed a significant increase in the macro structure of the right temporal lobe after music therapy intervention in the experimental group. Particularly, there was a noticeable trend of fiber bundle reconstruction in the internal sac and corpus callosum. In the experimental group, the increase in the FA value in the knee and pressure area of the corpus callosum was significantly correlated with the improvement in the GSC score. In addition, FA increased significantly in the posterior corpus callosum, corticospinal tract, cingulate tract, posterior branch of internal capsule, inferior fronto-occipital tract and superior longitudinal tract. Such findings positively correlated with increased blinking and motor reflex in the experimental group. In the speech reflex of GCS between the music group and the familial group, the difference between the two groups was not significant, but it was significantly different from the standard care group. This behavior result is also verified in the results of DTI imaging. It is well known that auditory semantic understanding is in the ventral pathway of the left temporal lobe, while speech output is in the temporoparietal and frontal lobe regions that transmit auditory-motor signals. Songs expressing emotion can be understood as an emotional language to some extent. When the vocal music is sung live, the right middle and front of the superior temporal gyrus, and amygdala of the subjects were involved in the activity at the same time. Therefore, in the experimental group, both the bilateral temporal lobe and the ventral frontal parietal cortex based on semantic understanding and melody perception responded simultaneously, forming an automatic processing of auditory integrity-verbal motor output, hence inducing the occasional verbal phonological behavior. However, in the familial group, because the conversational communication is more integrated the semantic understanding area of the left superior temporal gyrus and the verbal motor output area of the frontal parietal lobe, the relative voice behavior feedback was also seen in the GCS score.

Because MCS patients are consistently characterized by abnormal white matter signals, after 4 weeks of quantitative stimulation with two different sounds, music and family conversation, definite changes in nerve fiber bundles in the ascending reticular activating system (ARAS) were evident in the music group (Figure 4; EG: T2). The musical stimuli given had the following characteristics: (1) the patient was familiar with the past and had emotional involvement; (2) music therapists sang live and helped patients to feel present. ANS HRV response increment through the connectivity of up and down neural fiber bundles. Compared with the other two groups, the bulbar, pons, and midbrain of the experimental group showed an obvious thickening trend. It is suggested that under the input signal of vocal activity, the enhancement of important physiological activities such as cardiovascular and respiratory strengthens the lateral branches of sensory nerves connecting the lower corticospinal tract and the ascending medulla in the brainstem and diencephalon (dorsal encephalon nucleus), which enhances the Brownian motion trajectory of water molecules, and thus remodels the nerve fiber bundle in the brainstem and the distal sub cerebral. (DTI 3D Video in Supplementary material).

For patients with MCS, the challenge of awakening consciousness is a widespread issue. Live music therapy, a non-invasive treatment primarily utilizing auditory input to the receptors and created by a music therapist, promotes physiological arousal of the HPA axis and ARAS activation in MCS patients. Such innovative treatment approach aligns with the growing trend of heart-brain integrated prevention strategies and offers a new avenue for the clinical treatment of consciousness disorders.

## Limitations

The small sample size is one of the limitations of the present study. If a larger sample size can be included in future studies, so as to expand the number of subjects under three different conditions for comparison, there will be more evidence for clinical research.

### Implications for clinical practice

In the present study, the clinical significance of live music therapy administered by music therapists for patients with MCS was demonstrated, and the potential therapeutic mechanisms were tentatively revealed. In the future, the hope is that an increasing number of music therapists will participate in clinical practice as part of a multidisciplinary team, under the guidance of medical professionals, with the aim of aiding in the awakening of DOC patients.

## Conclusion

Live music therapy, administered by a professional music therapist, is more effective for MCS patients than listening to familial auditory stimulation, and is worth promoting in clinical practice in the future.

## Data availability statement

The original contributions presented in the study are included in the article/Supplementary material, further inquiries can be directed to the corresponding author.

## Ethics statement

The studies involving human participants were reviewed and approved by the Ethics Committee of China Rehabilitation Research Center (approval No. 2018–022-1) on March 12, 2018, and informed consent was obtained from the participants, relatives or guardians before commencing the study. The study trial was registered with the Clinical Trial Registry (Registration No. ChiCTR1800017809) on August 15, 2018. The patients/participants provided their written informed consent to participate in this study. Written informed consent was obtained from the individual (s) for the publication of any potentially identifiable images or data included in this article.

### Declaration of patient consent

The authors certify that they obtained consent forms from patients. In the form, patients gave their consent for their images and other clinical information to be reported in the journal. The patients understood that their names and initials would not be published.

### Reporting statement

The writing and editing of the article were performed in accordance with the Consolidated Standards Of Reporting Trials (CONSORT) Statement.

### Biostatistics statement

The statistical methods of this study were reviewed by the epidemiologist of Capital Medical University, China.

### Data availability statement

Deidentified participant data, along with corresponding data dictionaries, will be made available for sharing. Furthermore, related documents such as the study protocol and statistical analysis plan will also be accessible. The data will become available in the next 5 years. Research colleagues can access the data through the China Clinical Trials Registry, the Resman clinical trials public administration platform.

## Author contributions

XX was in charge of manuscript writing. XZ was responsible for study design, patient allocation, protocol development, data statistics, result description, statistical chart drawing, clinical analysis and discussion description, and is the corresponding author. WC implemented the assessment, evaluation, original data collection and supported music therapy. All authors contributed to the article and approved the submitted version.

## Funding

This work was supported by the Beijing Science and Technology Project Foundation of China, No. Z181100001718066, and the National Key R&D Program of China Nos. 2022YFC3600300 and 2022YFC3600305.

## Conflict of interest

The authors declare that the research was conducted in the absence of any commercial or financial relationships that could be construed as a potential conflict of interest.

## Publisher’s note

All claims expressed in this article are solely those of the authors and do not necessarily represent those of their affiliated organizations, or those of the publisher, the editors and the reviewers. Any product that may be evaluated in this article, or claim that may be made by its manufacturer, is not guaranteed or endorsed by the publisher.
